# Cross-orientation suppression in visual area V2

**DOI:** 10.1038/ncomms15739

**Published:** 2017-06-08

**Authors:** Ryan J. Rowekamp, Tatyana O. Sharpee

**Affiliations:** 1 Computational Neurobiology Laboratory, Salk Institute for Biological Studies, La Jolla, California 92037, USA; 2 Department of Physics, University of California San Diego, La Jolla, California 92093, USA

## Abstract

Object recognition relies on a series of transformations among which only the first cortical stage is relatively well understood. Already at the second stage, the visual area V2, the complexity of the transformation precludes a clear understanding of what specifically this area computes. Previous work has found multiple types of V2 neurons, with neurons of each type selective for multi-edge features. Here we analyse responses of V2 neurons to natural stimuli and find three organizing principles. First, the relevant edges for V2 neurons can be grouped into quadrature pairs, indicating invariance to local translation. Second, the excitatory edges have nearby suppressive edges with orthogonal orientations. Third, the resulting multi-edge patterns are repeated in space to form textures or texture boundaries. The cross-orientation suppression increases the sparseness of responses to natural images based on these complex forms of feature selectivity while allowing for multiple scales of position invariance.

## Abstract (web summary)

V2 neurons exhibit complex and diverse selectivity for visual features. Here the authors use a statistical analytical framework to model V2 responses to natural stimuli and find three organizing principles, chief among them is the cross-orientation suppression that increases response selectivity.

Object recognition relies on a series of complex and overall poorly understood transformations that ultimately give rise to our ability to recognize specific objects under continuous transformations, such as translation, scaling and rotation^1^,^2^. In the cortex this chain of transformations begins with the primary visual cortex (V1) where neural selectivity can be summarized as representing edges and bars of different orientation and position. This selectivity is sharpened by a variety of nonlinear suppressive mechanisms^3^,^4^,^5^,^6^,^7^, but the first-order responses to edges and bars provide a working framework within which to quantitatively study neural circuits in V1. Such a framework is missing for the next visual area V2 where one finds bewildering forms of feature selectivity compared to V1. There are multiple anatomical compartments^8^,^9^ each with different types of neuronal subpopulations^10^,^11^,^12^,^13^,^14^. Individual neurons in these subpopulations typically exhibit selectivity to multiple edges of different orientation and positions^12^,^15^,^16^, specific texture samples^17^,^18^ and texture boundaries^13^,^19^,^20^,^21^, as well as other higher-order patterns^19^,^22^,^23^. The increased complexity of V2 feature selectivity presumably requires similarly complex suppressive mechanisms to avoid confusion between different patterns. Previous studies point to the increased role of suppression in V2 compared to V1 (refs 14, 24) as well as in the area MT^25^. Yet, how suppressive mechanisms work in V2 to enhance the selectivity to more complex image features is not known. The problem is further exacerbated by the larger degree of position invariance in such neural responses^21^,^26^,^27^,^28^.

To address these questions of how feature selectivity in V2 is organized and sharpened by suppressive mechanisms, we developed a statistical framework for analysing neural responses to natural stimuli that brings together two long standing approaches in computational neuroscience: (i) analysis of multi-component feature selectivity using methods such as spike-triggered covariance^29^,^30^,^31^,^32^,^33^ and (ii) methods for analysing position invariant neural responses, such as convolutional models^25^,^34^,^35^,^36^,^37^,^38^,^39^. Applying this modelling approach to neural responses in the secondary visual area V2 to natural stimuli, we report here that (1) incorporating position invariance improves prediction accuracy on novel data sets, (2) multiple excitatory and suppressive features affect the responses of individual neurons, even after accounting for position invariance, (3) neurons form two classes based on diversity of orientation signals they encode, (4) excitatory and suppressive features pertaining to one neuron are arranged in an approximately orthogonal manner and (5) both excitatory and suppressive features form ‘quadrature pairs' that correspond to local position invariance. Overall, these findings show how nonlinear suppressive mechanisms can be incorporated into hierarchical signal processing schemes, similar to those proposed theoretically and used in computer vision algorithms^36^,^40^,^41^,^42^ in order to sharpen selectivity to complex image patterns in the presence of position invariance at multiple scales.

## Results

### Quadratic convolutional model

A tested way to find multiple relevant image features that may affect the neural responses is to expand the stimulus description from its D-pixel values to D+D^2^ values in order to include all pairwise products between the pixel values^29^,^30^,^31^,^32^,^33^. In the expanded stimulus space, one can compute the filter that, similarly to the spike-triggered average^33^,^43^, best accounts for the neural response (Supplementary Fig. 1). Because we are dealing with natural stimuli that have non-Gaussian statistics^32^,^44^,^45^, the relevant filter will be computed here by maximum likelihood optimization rather than simple averaging (see Methods). The resultant filter has two parts: a D-dimensional vector **v**^(1)^ that describes the single most relevant pattern in the original stimulus space and D^2^-dimensional filter *J* that represent the most relevant pattern in the quadratically expanded space *s*_i_*s*_j_ (*s*_i_ are pixel values or other stimulus components). This second part *J* of the filter can be transformed into a square matrix and diagonalized to yield a set of relevant input dimensions^32^. The resultant dimensions either directly correspond to the relevant image features for a particular neuron or comprise their linear combinations. It is also noteworthy that the modelling framework can detect relevant features even if they affect the neural responses only through higher than second-order interaction. For example, in Supplementary Fig. 4 we show that it is possible to find relevant features of a model neuron whose responses are based on a third-order conjunction between the relevant features. The reconstruction becomes possible because third- and higher-order interactions can be approximated as combinations of multiple pairwise interactions, as has also been demonstrated for human perception^46^,^47^.

**Figure 1:**
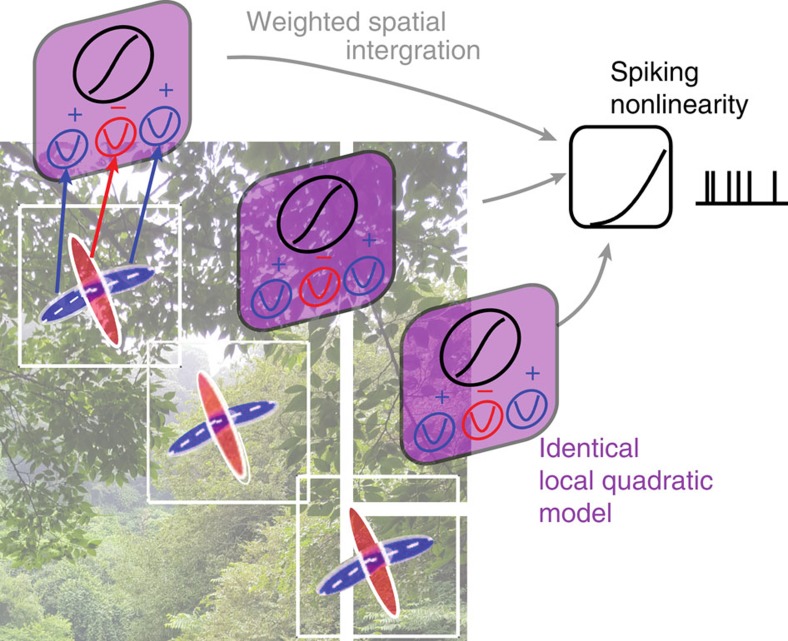
A schematic representation for the QC model. Each input image is split into overlapping patches. Stimuli from within each patch are passed through a set of linear filters, followed by quadratic nonlinearity (with a linear term). The squared filter outputs are added together with different weights. The weights are positive for excitatory subunits (blue Gabor outlines) and negative for suppressive subunits (red Gabor outlines). Applying logistic transformation yields the response of each QLS. The QLS parameters are the same for all patches. A weighted sum of the QLS outputs across time and spatial patches describes temporal dynamics and graded position invariance, respectively. A rectifying nonlinearity applied to the weighted sum of the QLS outputs yields the predicted firing rate. QLS, quadratic logistic subunit.

Here, we combine the approach of minimal quadratic models with ideas of methods designed to describe graded position invariance^25^,^34^,^35^,^36^,^37^,^38^,^39^. Specifically, we apply the quadratic stimulus transformation not to the whole stimulus at once, but separately to the overlapping patches that together cover the full image (Fig. 1). Extending the weighting function to different latencies before the spike^25^, the approach can also take temporal dynamics into account.

The overall model, to which we refer as the quadratic convolutional (QC) model, has three nonlinearities (Fig. 1): (i) the quadratic function that is applied locally to image patches and which provides a good description of V1 complex cell responses^14^,^29^,^31^,^48^,^49^,^50^; (ii) the sigmoidal function applied after pooling across all ‘complex cell' subunits within a given patch, that is, at one position in the visual field; and (iii) the final rectifying nonlinearity to produce positive signals suitable for comparison with the neural spike rate. Removing the quadratic nonlinearity reduces the method to the convolutional model, where one seeks to account for neural responses using the same feature shifted to different positions^34^,^35^. On the other hand, removing the sigmoidal nonlinearity reduces the present model to the kind described by the spike-triggered covariance and related methods^29^,^30^,^31^,^32^,^37^ where all relevant features are found without taking into account position invariance. Taking position invariance into account reduces the overall number of features that need to be estimated, which results in models that can be interpreted better^34^,^35^,^51^ and which are likely to yield higher predictive power on novel data sets^34^,^35^.

**Figure 2:**
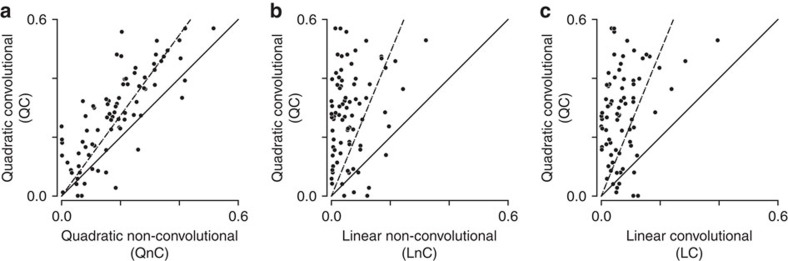
QC model outperforms models without either position invariance or multi-component feature selectivity. Extrapolated correlation between model predictions for a novel test set and the observed responses^64^ is shown in all panels. The dashed lines indicate the linear regression without a constant offset. (**a**) The QC model outperformed the QnC; average improvement is by a factor of 1.5. (**b**,**c**) QC also outperformed an LnC and the LC by factors 3.9 and 4.4, respectively. LC, linear convolutional model; LnC, linear non-convolutional model; QnC, quadratic non-convolutional.

After testing the optimization algorithm on model neurons (Supplementary Note 1 and Supplementary Figs 1–4 and Methods), we fit the model parameters to V2 neural responses to natural stimuli. The results indicate that the full QC model yielded better predictions compared to reduced models where either quadratic or sigmoidal nonlinearities have been removed (Fig. 2). On an average, the QC model outperformed the linear convolution model by a factor of 3.9. This indicates a strong impact of multi-component feature selectivity on neural responses. The incorporation of position invariance also had a strong impact, because the QC model on average performed 50% better than the quadratic non-convolutional model. The QC model also outperformed the standard linear-nonlinear model that accounts for neural responses based on a single relevant image feature followed by a nonlinearity^33^,^52^. In what follows, we refer to this latter model as linear-non-convolutional because it uses a specific nonlinearity in common with quadratic non-convolutional and QC models. We also found that an alternative model structure with a logistic final nonlinearity instead of the soft-plus function resulted in systematically worse performance for all models (*P*<0.01), Supplementary Fig. 5. To summarize, both position invariance and selectivity to multiple image features are necessary to account for the responses of V2 neurons.

### Excitatory and suppressive features of V2 neurons

**Figure 3:**
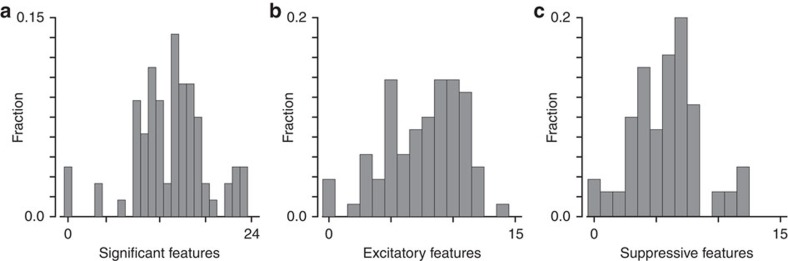
The distribution of the number of relevant image features that affect V2 responses after factoring out position invariance. (**a**) The total number of significant dimensions of *J* (features, 13.4 on average). (**b**,**c**) The distribution of excitatory (mean 7.6) and suppressive (mean 5.8) features. All distributions are non-unimodal with *P*=0.0145, 0.0036, 0.0003, respectively, for the Hardigans' dip test, *N*=80.

Even after factoring out position invariance, the responses of V2 neurons could not be described by a single template and instead required the presence of multiple relevant image features. For the vast majority of neurons, the eigenvalue of analysis of kernel *J* identified the presence of both multiple excitatory and multiple suppressive features (excitatory and suppressive features correspond to eigenvectors of *J* with positive and negative eigenvalues, respectively, see Methods). On average, there were 7.6 excitatory and 5.8 suppressive features. Echoing previous results^14^, the distribution of suppressive features was non-unimodal, as were the distributions of excitatory features and the distribution of the total number of relevant features per neuron (Fig. 3). The total number of relevant features is the total number of significant eigenvectors of *J*. The number of excitatory and suppressive features per neuron were strongly correlated (*P*=0.016, *t*-test, two-sided, *n*=80), indicating that the complexity of excitatory and suppressive signals co-vary together.

The non-unimodal aspects of the distribution of the number of features relevant to the responses of individual V2 neurons suggest the presence of separate populations of V2 neurons. To understand how these classes might be related to those identified previously among V2 neurons^10^,^11^,^12^,^14^ and what signals V2 neurons from each class represent, we fit the set of relevant image features for each neuron as a combination of Gabor patterns. This approach makes it easier to interpret the reconstruction results in terms of putative inputs from V1 (refs 10, 11, 14). The excitatory and suppressive features were fit separately to yield as close as possible match to the *J* kernel of the model (Methods). For all neurons, this resulted in statistically significant correlations between the *J* kernel and its fit in terms of combinations of Gabors (Supplementary Fig. 8). The fit yields not only a set of relevant Gabors for each neuron but also the weights that characterize how strongly the neuron's firing rate is affected by a given Gabor. The weights are positive for excitatory and negative for suppressive Gabors.

**Figure 4:**
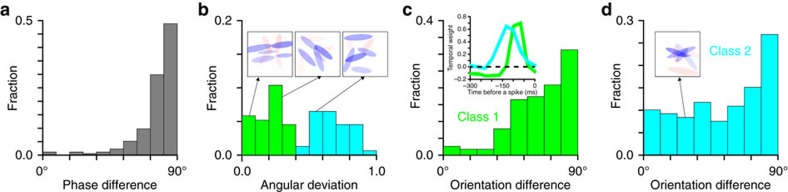
Organization of excitatory and suppressive features for two subpopulations of V2 neurons. (**a**) Spatial phase difference between the nearest-neighbor excitatory Gabors shows a peak at 90°, which is consistent quadrature pairing and local position invariance. (**b**) The angular standard deviation of the excitatory Gabors for each neuron. The neurons separate into two classes: one with similar orientations ‘uniform' and one with diverse orientations ‘nonuniform'. These neurons also have different dynamics, biphasic for class 1 and unimodal for class 2 (**c**) inset. The distribution of nearest-neighbour differences between excitatory and suppressive features for the ‘uniform' (**c**) and ‘nonuniform' (**d**) class. (inset, **b**,**d**) Examples of the Gabors fit to neurons from each class. Blue and red denote excitatory and suppressive features, respectively; opacity is proportional to the weight of the Gabor feature.

Analysis of the sets of relevant Gabors yielded three observations. First, both excitatory and suppressive Gabors formed ‘quadrature pairs'^48^,^49^. Within the pair, the two Gabors have all of the same parameters except for the spatial phase (Fig. 4a). For the vast majority of neurons, the spatial phase was offset by a value close to 90°. This is the same phase difference as between a sine and a cosine Gabor. A sine Gabor can be used to describe an edge, whereas a cosine Gabor describes a bar. Together, these two features describe invariance to small shifts in direction perpendicular to the edge/bar. This type of pairing has been shown to describe well responses of complex cells in V1 (refs 48, 49) and therefore can help interpret the present results in terms of V1 inputs to V2. On the basis of the observation of quadrature paring between Gabor features, we refitted *J* kernels directly as arising from combinations of different Gabor quadrature pairs. This resulted in almost no decrease in fit quality (Supplementary Fig. 8) despite having less than half as many parameters.

Analysis of excitatory Gabor pairs has revealed two subpopulations based on the variance of relevant orientations for each neuron (Fig. 4b, *P*=0.0061 Hartigans' dip test, *n*=77). Neurons in the first class had smaller variance across orientations compared to neurons from the second class. For neurons in the first class, all excitatory orientations typically form one or several smooth curves of similar orientation (see Fig. 4b insets for examples). In contrast, neurons in the second class often had a fan-like pattern of Gabor features. Following ref. 12, we refer to neurons in the first class as ‘uniform' and neurons in the second class as ‘non-uniform'. This classification also connects with previous classifications of V2 neurons into ‘transient' and ‘sustained' classes based on response dynamics^11^,^13^,^20^, because we found uniform neurons to have biphasic temporal kernels whereas non-uniform neurons had unimodal (Fig. 4c, inset) temporal kernels.

For V2 neurons from both classes, excitatory and suppressive Gabors formed an orthogonal pattern. The effect was more pronounced for neurons in the ‘uniform' class (Fig. 4c) compared to neurons in the non-uniform class (Fig. 4d). Partly this is due to the fact that ‘non-uniform' neurons often had a fan-like pattern (for example, inset in Fig. 4d). The presence of rapidly changing excitatory orientation at nearby positions in such a pattern can cause a suppressive Gabor that is orthogonal to one excitatory Gabor to not be orthogonal to other nearby excitatory Gabors. Nevertheless the bias towards orthogonality was statistically significant for both neuron classes (*P*<10^−13^ and *P*<0.0005, respectively, *χ*^2^-test against uniform distribution with seven degrees of freedom). Further, the trend persisted even when classes are combined (Supplementary Fig. 9a) and when the analysis is expanded to include all Gabor pairs beyond the nearest neighbours (Supplementary Fig. 9b). We also note that the incidence of iso-oriented suppression, while small, was larger for excitatory-suppressive pairs that are not nearest neighbours.

### Spatial pooling and texture selectivity

**Figure 5:**
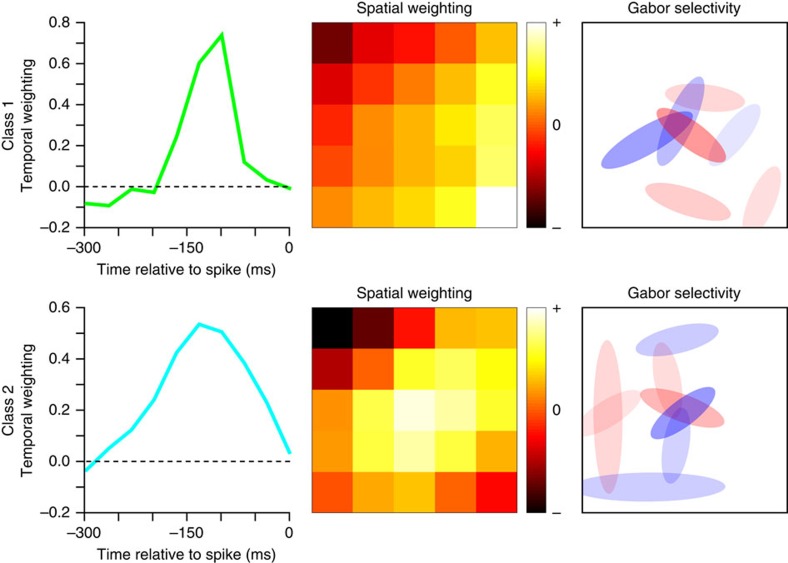
Two example neurons with biphasic spatial pooling. (top) Example neuron from ‘uniform' class. (bottom) Example neuron from ‘nonuniform' class. For each neuron we show its temporal filter, spatial pooling mask and the set of excitatory and suppressive features using the same format as insets in Fig. 4b.

The excitatory and suppressive features discussed so far describe neuronal selectivity at one spatial position. The last component of the model—the spatial pooling mask **v**^(2)^—describes how signals these signals are combined across space. For most neurons (66) the spatial pooling was approximately uniform. An example neuron with such type of pooling is provided in Supplementary Fig. 6. The uniform pooling yields selectivity to a patch of texture that is defined by the observed combination of relevant excitatory and suppressive features. In addition to uniform pooling, we also observed biphasic pooling in approximately 34% (26/77) of neurons. Examples of neurons from class 1 and class 2 with biphasic pooling masks are shown in Fig. 5. This type of pooling is notable because it can mediate texture segmentation^13^,^20^,^21^,^53^, a function that has received a lot of attention in V2 (refs 11, 13, 20, 21). The incidence of biphasic pooling was 25% (10/40) for ‘uniform' class 1 neurons and 45% (16/37) for class 2 ‘non-uniform' neurons.

### Cross-orientation suppression increases response sparseness

**Figure 6:**
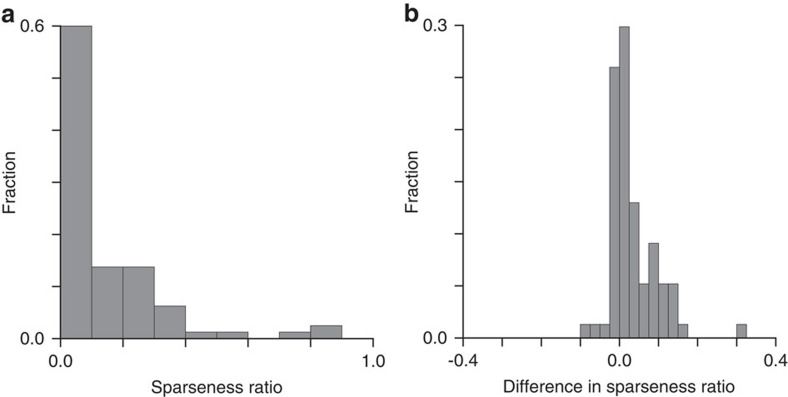
Suppressive features strongly increase the sparseness of neural responses. (**a**) The fraction of sparseness that remains after suppressive features are removed is plotted as a distribution across the data set of V2 neurons. Sparseness is computed across all natural image inputs presented to a given V2 neuron. (**b**) Cross-orientation suppression increases the sparseness of neural responses. Difference in sparseness ratio between the model based on all relevant Gabor features compared that where orientation of suppressive Gabors have been set at random.

What is the functional significance of suppressive features? In V1, cross-orientation suppression leads to sharper orientation tuning and sparser responses^3^,^4^,^5^,^6^,^7^. The sharpness of orientation tuning would not be an appropriate measure in V2 given the increased complexity of relevant features. However, we can evaluate the impact of suppressive features on the sparseness of responses by comparing the sparseness of predicted responses with and without the suppressive features. We find that suppressive features have a dramatic effect on sparseness, increasing the sparseness by a factor of >8 (Fig. 6a). Further, to evaluate the impact on sparseness of specific orientation differences between excitatory and suppressive features, we compared the sparseness of models based on estimated relevant Gabor features with that of models where orientation of suppressive Gabors were chosen at random. The decrease in sparseness was systematic and highly statistically significant (*P*=5 × 10^−6^, Wilcoxon signed rank test, two-sided, *n*=77), Fig. 6b.

## Discussion

The secondary visual area V2 is notorious for complexity of its organization. This includes the presence of multiple anatomical compartments^8^,^9^ as well as diversity in the types of inputs it receives from V1 (ref. 54), orientation selectivity properties^10^,^12^,^14^,^55^, temporal dynamics^11^,^13^ and the suppression strength^14^. Here we used statistical analysis of neural responses to natural stimuli to find several organizational principles that could help systematize and understand the complexity of V2 responses.

A number of prior analyses of V2 responses have indicated the presence of two or more subpopulations^10^,^11^,^12^,^13^,^14^,^20^,^21^. The two classes of V2 neurons that we identify here are most directly analogous to uniform and non-uniform selective neurons^12^ and to the ‘ultralong Gabor' and ‘complex-shaped' neurons^10^. It has been proposed that ‘uniform' or ‘ultralong Gabor' neurons should correspond to the ‘transient' subpopulations identified based on temporal integration properties^11^. Our finding that ‘uniform' neurons indeed have biphasic temporal response kernels whereas ‘non-uniform' neuron have integrative temporal properties provides support for this hypothesis, thus helping to connect different studies of V2 subpopulations.

One advantage of the statistical analysis carried out here is that it can pick up slight differences in the preferred orientations at nearby positions. Therefore, we find that even neurons that may be classified as ‘uniform' or ‘ultralong', have multiple excitatory Gabors at slightly different orientations at different positions that together would form a line of noticeable curvature (Fig. 4b insets). While the spread of orientations for ‘uniform' neurons is nonzero, there is still a clear break in the distribution of orientation spreads that separates ‘uniform' from ‘complex-shaped' neurons (Fig. 4b). This then provides further justification for the separation of neurons into the two classes.

Because the QC model explicitly separates position invariance from complexity of feature selectivity, some of the complex-shaped neurons that have position invariance, such as complex-unoriented^26^ and spot stimuli^27^, can now be described using a smaller number of features. Incorporation of position invariance also improves predictive power on novel data sets compared to the model with no position invariance^10^, on average by a factor of 1.5. While the correlation numbers are lower than those have been recently reported in higher visual areas such as V4 (refs 36, 56), we note that here the predictive power is computed with an explicit model that has a fixed nonlinearity rather than up to an arbitrary one-to-one nonlinearity^56^ or a linear^36^ transformation. The QC model also directly informs our understanding of the feature selectivity, which is difficult to do in models based on deep networks^36^,^57^.

Neurons from the primary visual cortex project to both V2 and area MT. Whereas neurons projecting to area MT have consistent visual response characteristics^58^, projections to V2 include in approximately equal proportions neurons that are invariant and selective for spatial phase^54^. We find that both excitatory and suppressive Gabor features for one V2 neuron form quadrature pairs, and thus occur in combinations that are invariant to spatial phase. It is possible that strong V1 input that is dominated by one spatial phase is taken into account by the linear part of the quadratic model instead of the quadratic part. However, we find that linear convolutional models yield much worse predictive power than QC models, on average by a factor of 4 (Fig. 2). These observations then suggest that when V1 neurons that are sensitive to spatial phase project to V2 they do so together with other V1 neurons tuned to other spatial phases with similar orientation/position. Collectively, the contributions from these V1 neurons would then yield to good descriptions by models invariant to spatial phase.

We find that excitatory and suppressive signals are organized locally according to the principles of cross-orientation suppression^3^,^4^,^5^,^6^. This finding brings considerable simplification to models of suppressive mechanisms in area V2. Previous studies using gratings in V2 did not probe suppressive mechanisms at the same position as excitatory signals^12^,^13^. Especially in the case of ‘non-uniform' neurons, the complexity of orientation tuning across positions makes it difficult to systematize the contribution of suppressive mechanisms. Long records of neural responses to natural stimuli contain this information because of the variety of edge combinations and other higher-order statistics^45^,^47^,^53^ present in the natural scenes. Extracting this information by statistical analysis reveals a surprisingly simple pattern of excitatory and suppressive signals employed by V2 neurons: even if excitatory signals are organized in a complex manner, suppressive signals work locally to enhance representation of excitatory signals through cross-orientation suppression.

In addition to local cross-orientation suppression, we also detect two kinds of iso-orientation suppression acting on broader spatial scales. The first kind of iso-orientation suppression corresponds to those few cases where excitatory and suppressive Gabors had similar orientations (Supplementary Fig. 9). The incidence of such cases, while small, was greater for Gabors that are not nearest neighbours. On the basis of this, we identify this iso-orientation suppression as most likely originating from the surround of V1 receptive fields^20^,^59^,^60^. The weakness of this type of iso-orientation suppression in this experiment could in part be due the slower dynamics of surround integration compared to suppression from within the receptive field center^61^,^62^.

The second kind of iso-orientation suppression is represented by biphasic spatial pooling in the second layer of the model. This type of subtractive interaction can aid detection of borders defined by changes in texture^20^,^21^,^53^. The biphasic pooling was prominent in the data set (34% of neurons), with uniform pooling observed in the remaining cases. Overall, the observed patterns of selectivity based on locally orthogonal excitatory and suppressive features that are repeated across a range of spatial position could mediate the observed selectivity of V2 responses to textures^17^,^18^ and texture boundaries^13^,^20^,^21^.

## Methods

### Electrophysiological recordings

We applied our method to a data set of neural recordings from visual area V2 that was previously published^14^ and available through the CRCNS data sharing website^63^. The data set included recordings from three awake and behaving male rhesus macaque monkeys. Detailed methods on electrophysiology are in ref. 14. Briefly, during the recording, the animals performed a fixation task for a juice reward. The stimulus was a series of patches from greyscale images presented rapidly for 3–5 s trials. The image patches were scaled to be 2–4 the size of the classical receptive field (as determined using reverse correlation with a dynamic sparse noise stimulus).

### Quadratic convolutional model

The model seeks to predict a neuron's response Y_t_ (measured as a number of action potentials/spikes) given the stimulus **X**_t_ presented to the animal. The first step of the model is to convolve the identical subunits with the stimulus, which is equivalent to extracting patches **x**_i,t_ from the stimulus from different positions and times. The patches are passed through logistic subunits with identical quadratic filter *J*, linear filter **v**^(1)^, and bias *a*^(1)^ to produce the first layer's response


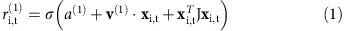


where σ(x) is the logistic function


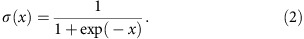


To take into account the possibility of the overall position invariance, the responses of the quadratic logistic subunits are pooled using the weights **v**^(2)^ and rectified


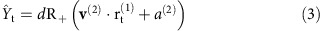


where *d* is a scaling factor, *a*^(2)^ is another scalar bias, and R_+_(x) is the soft-plus rectifier


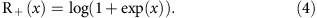


All of the parameters of the model (*a*^(1)^, **v**^(1)^, *J*, **v**^(2)^, *a*^(2)^, *d*) were fit by minimizing the Poisson negative log-likelihood


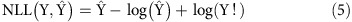


using stochastic gradient descent. For our analysis of V2 data, stimuli were 20 × 20-pixels by 10 frames in time, binned at 16 ms. The patches **x** were 16 × 16-pixels by one frame, making **v**^(1)^ a 256-dimensional vector, and *J* a 256 × 256 matrix. The offsets in pixel-space were 1-pixel, which produced a 5 × 5 spatial grid for pooling responses of the quadratic subunits defined by equations (1) and (2). We also used 10 latencies to account for the neural response dynamics, which in total results in 5 × 5 × 10=250 dimensional vector **v**^(2)^.

We divided the training data into fourths and used three-fourths to calculate the gradient and one-fourth as a validation set to determine when to stop training. For each neuron, we calculated four models each with a different fourth of the data as its validation set (Supplementary Fig. 7). The four models for each neuron were averaged, while variability between these can be used to gauge the impact of noise variability and stochasticity in the optimization process on model parameters.

The data set also included a separate set of responses to different repeated movies. Those data were used to evaluate model performance and are separate from the data used for fitting and early stopping. The model performance was evaluated in terms of correlation coefficients between measured and predicted responses after compensating for finite-size effects in the data^64^.

### Eigenvector significance

To determine which eigenvectors of *J* were significant, we generated shuffled *J* matrices to determine the distribution of maximum and minimum eigenvalues. We began by subtracting the mean of *J* to avoid the spurious eigenvalues that can be caused by a nonzero mean of *J* (ref. 65). We then randomly shuffled the diagonal and off-diagonal elements separately to create random symmetric matrices and build distributions of maximum and minimum eigenvalues. We then checked the eigenvalues of the zero mean *J* matrix in order of decreasing magnitude against the distributions to determine the probability to see a value of that magnitude in a random matrix. If the probability was <0.05, we considered the eigenvalue and the corresponding eigenvector to be significant.

### Fitting gabors

To characterize the feature selectivity of *J*, we approximated it using the weighted sum of Gabor wavelets. The equation for the Gabor wavelet is


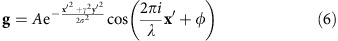


where


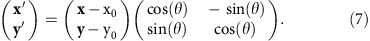


x_0_ and y_0_ indicate the location of the Gabor wavelet in the image, *θ* controls the orientation, *γ* controls the aspect ratio, *σ* controls the size of the Gabor, *λ* controls the spatial frequency, and *φ* controls the spatial phase. *A* is a normalization constant. With a set of Gabor wavelets **g**_i_ and corresponding weights *w*_i_, an approximation of *J* can be constructed from the weighted sum


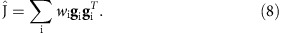


We fit two variations of this model. First, we fit the *J* matrix as representing independent Gabors, with the number of Gabors equal to the number of significant features. After finding that the Gabors form quadrature pairs (Fig. 4) we performed a second fit using pairs of Gabors with identical parameters except for φ, which was 0 and πi/2 in order to form a quadrature pair.

We fit the parameters of the Gabor wavelets using differential evolution^66^. We randomly generated 10 *P* sets of parameters where *P* was the number of parameters of the model. Parameters *x*_0_ and *y*_0_ were selected uniformly within limits set by the size of the frame. *θ* was selected uniformly from −π to π. σ, γ and λ were selected log-uniformly. In each iteration, new sets of parameters were generated using a combination of the existing sets using the differential evolution variant termed ‘rand/2/bin':


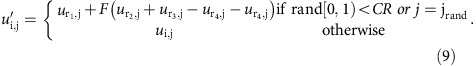


Here, r_1_ through r_5_ are the indices of five unique parameter sets excluding i. j_rand_ is randomly selected from {1,...,P} to ensure *u'*_i_ differs from *u*_i_ for at least one parameter. *CR* and *F* were initialized to rand[0,1) and 0.1+0.9 rand[0,1), respectively, for each parameter set and regenerated before generating **u′** if rand[0,1)<τ. τ was set to 0.1 for both *CR* and *F*. The multiplicative parameters σ, γ and λ were passed through equation (9) as their logarithms make the steps be changes in scale. The parameters were bound by their initialization ranges. If a new value was outside of those ranges, it was considered to have infinite error. The new set of parameters **u**'_i_ replaced **u**_i_ if the mean square error was lower. We ran the algorithm until none of the groups of parameters changed during an iteration. We ran multiple iterations of the algorithm with different initializations and selected the parameters with the lowest error.

The excitatory and suppressive parts of *J* were fit separately to minimize the mean squared error with the reduced *J* matrix constructed from only the significant excitatory or suppressive eigenvectors.

To determine whether the Gabors formed quadrature pairs, we paired the individual Gabors with similar positions and orientations and measured the difference in phase according to


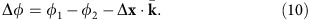


Δ**x** is the difference in the position of the Gabors, and 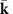 is the mean spatial frequency.

It is important to note that, although the quadratic subunit model of equations (1) and (2) accounts for neural responses based on combinations of pairwise features, this model can also identify features that affect the neural responses through higher than second-order interaction. To illustrate this, we applied the minimal quadratic model analysis^32^ based on the equations (1) and (2) to the responses of a model neuron that was exclusively sensitive to a third-order interaction between three relevant features^53^,^67^


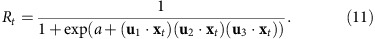


where **u**_i_ are three relevant features shown in the top row of Supplementary Fig. 4. The reconstructed features yield the subspace projection of 0.6 with the model features even though the form of the model neuron is different from the form of the model fit to it. The precise level of reconstruction accuracy depends on the strength of higher-order interactions as well as the correlation between second- and higher-order interactions. Given that in natural scenes correlations of different orders are correlated^44^,^46^,^47^, this illustration show performances in close to worst-case-scenarios for reconstructing higher-order features.

In Supplementary Fig. 6, we show all of the analysis steps for the example V2 neuron. The weighting mask includes both spatial and temporal components that can be separated using singular-value decomposition. The spatial component shows a preferred location with the response decreasing as the stimulus moves from that location. The temporal component shows a preference for stimuli ∼33 ms before the spike. If the preferred stimuli are present at longer latencies, the neuron's response would be suppressed. The linear kernel (b) shows a preference for horizontal bars. The quadratic kernel is difficult to interpret and requires decomposition into its linear components, which we describe next.

The eigenvalue analysis for this neuron's *J* kernel indicated the presence of 10 excitatory (c) and eight suppressive features (d). Therefore, the positive and negative part of the *J* kernel were fit using 10 and eight Gabors, respectively. The result of this fit is shown in the top row of c and d. The excitatory Gabors indicate selectivity to horizontal lines of varying spatial frequency. The middle row shows these Gabors projected into the dominant excitatory eigenvectors. The close correspondence between this row and the row above indicates that they are a good approximation of the neuron's selectivity. The bottom row shows the dominant excitatory eigenvectors of *J*. The broad selectivity for horizontal lines is evident, but the orthogonality of the eigenvectors obscures the underlying Gabor structure. All rows are arranged such that the features with the largest weight are on the left. Analysis of the suppressive feature reveals selectivity to predominantly vertical Gabors. Thus, excitatory and suppressive Gabors form an orthogonal pattern. To get a sense for the reproducibility of results, in Supplementary Fig. 7 we show results for different subsets of the data sets. The consistency of results across different subsets of the data can be used to gauge stability of results against neural noise and stochasticity during model optimization.

Because both models based on soft-plus rectifying nonlinearity as in equation (4) and a saturating nonlinearity as in equation (2) have been used to analyse properties of extra-striate visual neurons, we have compared the performance when either of these nonlinearities is used in place of the final nonlinearity equation (4). We find that for this data set the soft-plus function produced systematically better predictions of the neural responses on novel data subsets compared to the model with a saturating final nonlinearity (*P*<0.01, Wilcoxon signed rank test, two-sided, *n*=80, for all pairwise comparisons in Supplementary Fig. 5).

### Code availability

The code is available at https://github.com/rjrowekamp/quadratic-convolution.

### Data availability

The data sets analysed during the current study are available at the Collaborative Research in Computational Neuroscience (CRCNS) data sharing website, http://crcns.org/data-sets/vc/v2-1.

## Additional information

**How to cite this article:** Rowekamp, R. J. & Sharpee, T. O. Cross-orientation suppression in visual area V2. *Nat. Commun.* **8,** 15739 doi: 10.1038/ncomms15739 (2017).

**Publisher's note:** Springer Nature remains neutral with regard to jurisdictional claims in published maps and institutional affiliations.

## Supplementary Material

Supplementary Information Supplementary Figures, Supplementary Note and Supplementary Reference.
