# Supplementary material for: Cross-orientation suppression in visual area V2
Source: Nat Commun. 2017 Jun 8;8:15739. doi: 10.1038/ncomms15739 (PMC5472723; doi:10.1038/ncomms15739)
Supplement: Supplementary Information — Supplementary Figures, Supplementary Note and Supplementary Reference. [file ncomms15739-s1.pdf]

Supplementary Figures

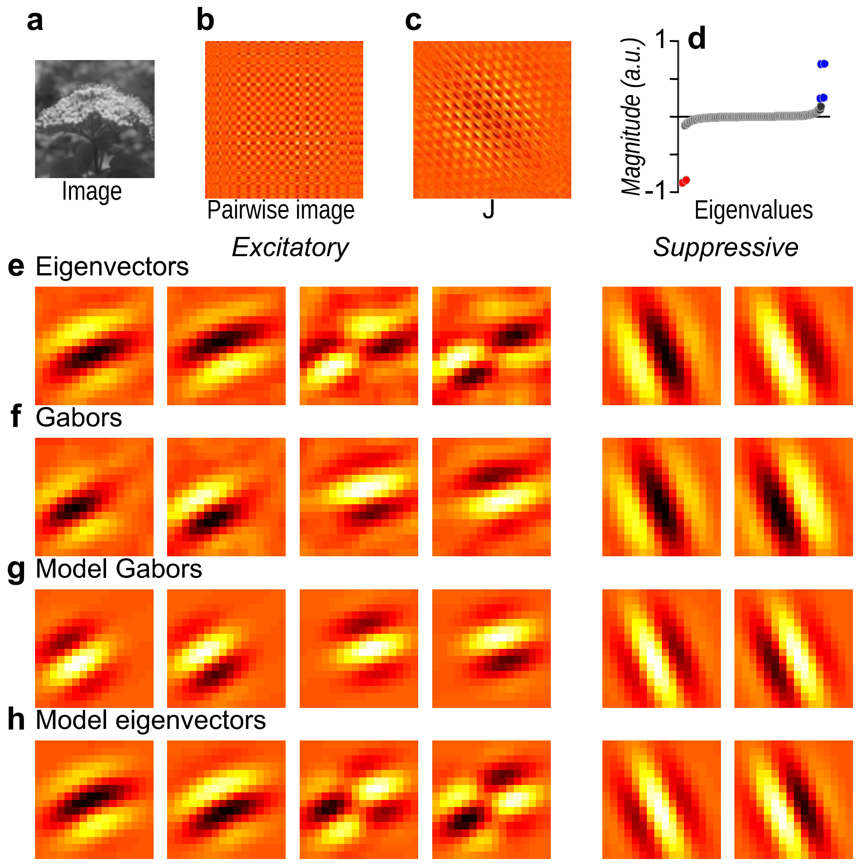

Supplementary Figure 1: **Demonstration and test of the quadratic convolutional algorithm on model neurons. a.** The algorithm begins with an image patch as the stimulus. **b.** The image is expanded into its pairwise values. **c.** The pairwise image is compared with the quadratic kernel J to determine the subunit response. The algorithm optimizes J to improve the similarity between the predicted and observed responses. **d.** Eigenvalues of J showing four positive and two negative statistically significant eigenvalues. **e.** The eigenvectors associated with the six significant eigenvalues. The eigenvectors associated with positive eigenvalues are excitatory, and those with negative eigenvalues are suppressive. **f.** Because the eigenvectors are orthogonal, they can obscure the underlying structure. Fitting J with Gabor pairs recover the model Gabor features from panel **g**. **h.** The model Gabors in the orthogonal representation.

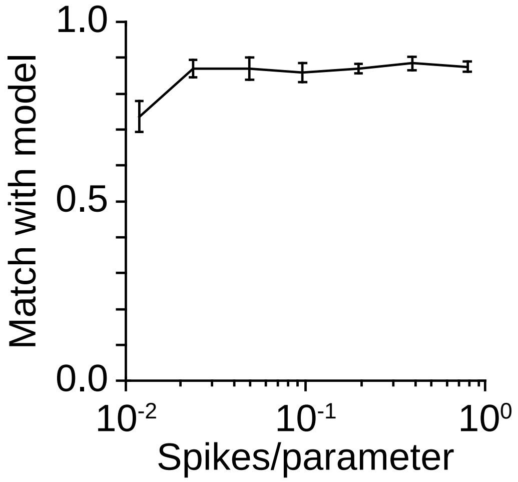

Supplementary Figure 2: **Underlying feature selectivity can be recovered even when the number of spikes is much less than the number of parameters.** Match between features used to generate spikes and the dominant eigenvectors of the J matrix as measured by subspace projection[1]. The match remained high even with less than 0.1 spikes/parameter. Simulated neuron used six features previously shown. The number of spikes/parameter was adjusted by shifting the scaling constant *d*.

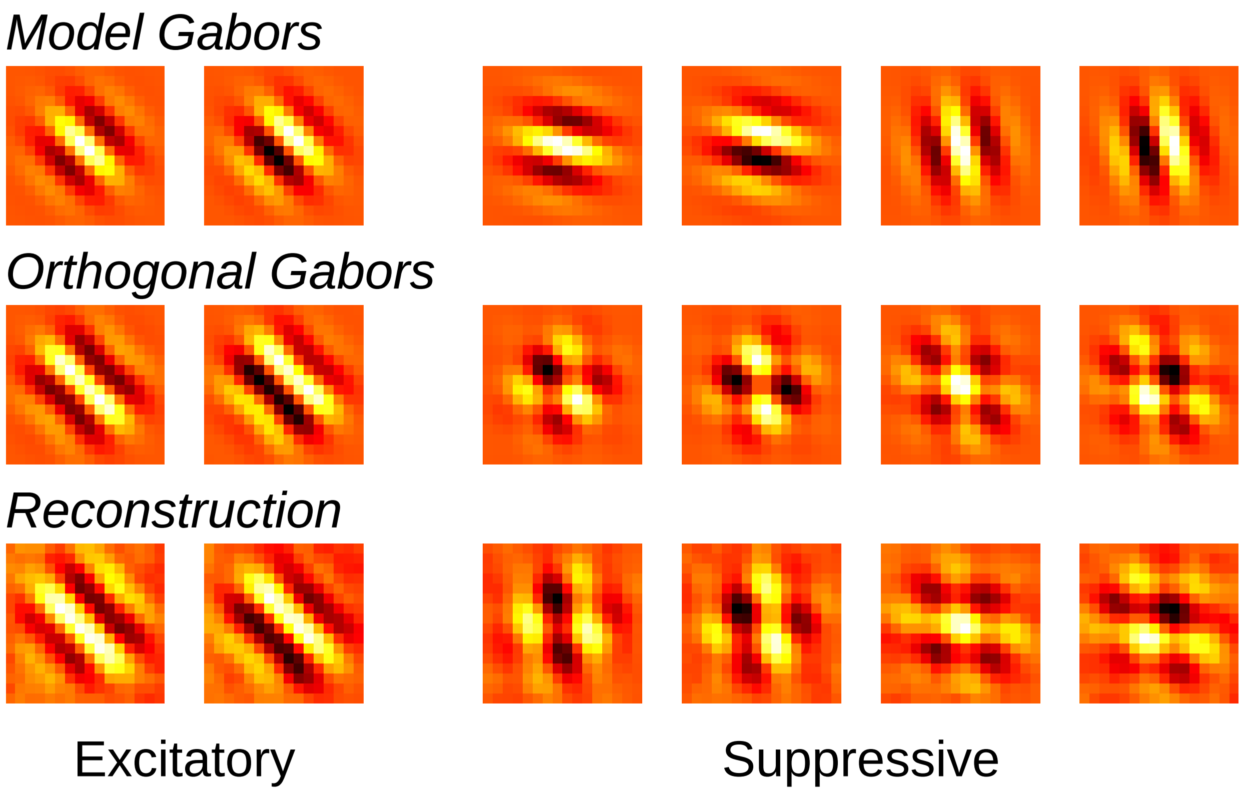

Supplementary Figure 3: **Cross-orientation suppression is not an inherent property of the reconstruction algorithm.** Here, the algorithm is applied to a model neuron whose excitatory (first four columns) and suppressive (last two columns) features had similar and not orthogonal orientations. The reconstruction (bottom row) yields a subspace projection value of 0.957 compared to model features (top row; middle row: orthogonal representation of model features).

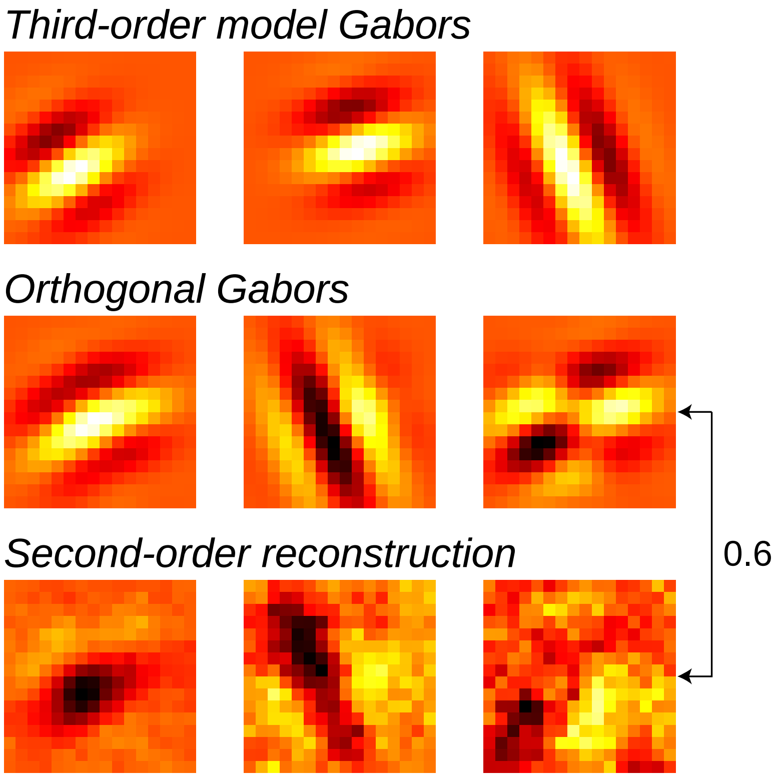

Supplementary Figure 4: **A demonstration that higher-order features can be reconstructed using combinations of multiple pairwise interactions.** Top row shows three relevant features. Middle row shows the relevant features in the orthogonal representation, bottom rows shows the top three eigenvectors. The reconstructed features yield subspace projection of 0.6 with the model dimensions.

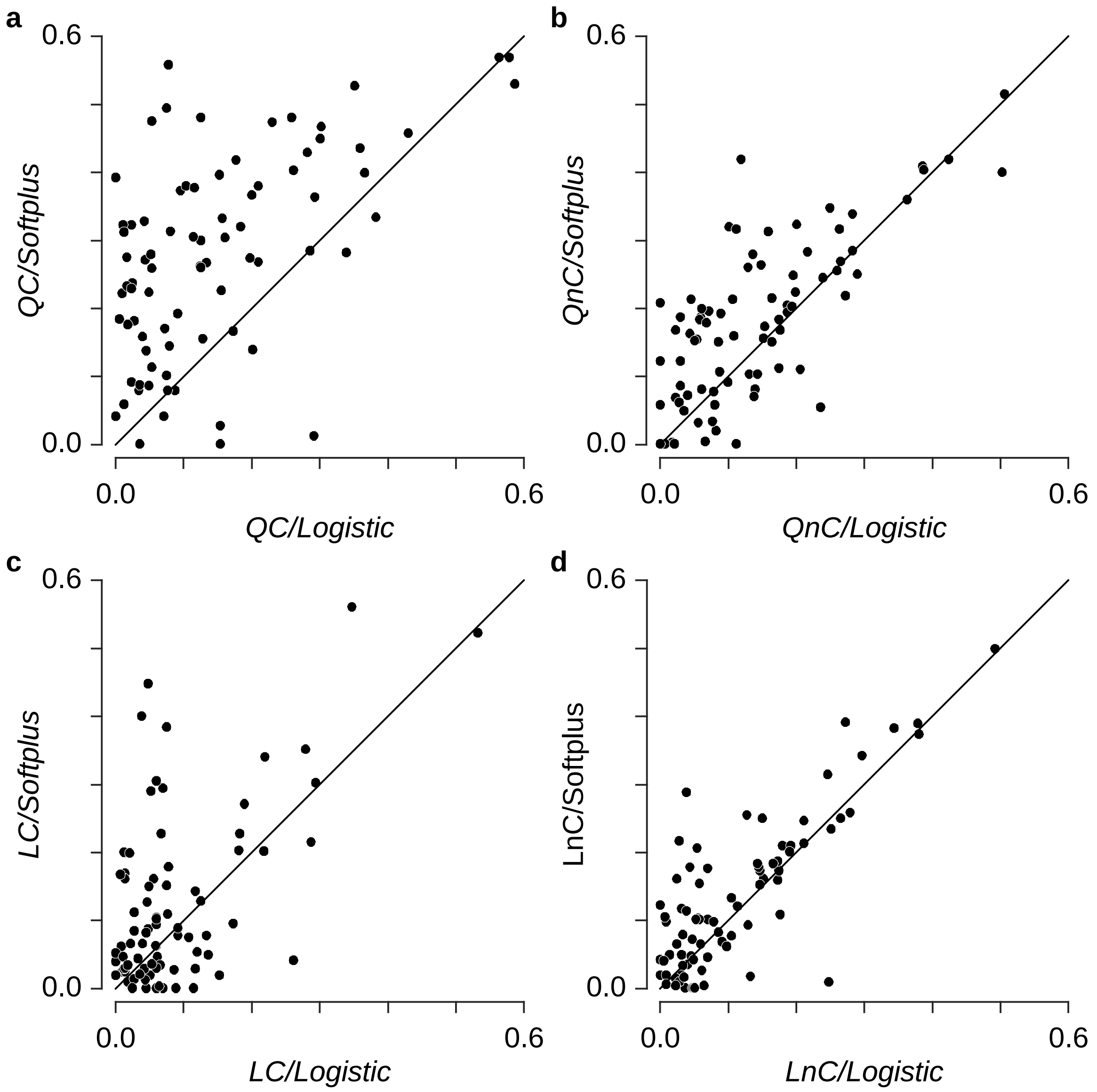

Supplementary Figure 5: **Softplus rectifier nonlinearity yields better predictive power than models with logistic function for second layer nonlinearity.** Models using a softplus rectifier have higher correlations with observed responses from held out test set than equivalent models with a logistic function as the second layer nonlinearity. (Wilcoxon signed rank test, two-sided, $n = 80$, quadratic invariant $p < 10^{-10}$, quadratic non-invariant $p < 10^{-3}$, linear invariant $p < 10^{-2}$, and linear non-invariant $p < 10^{-2}$).

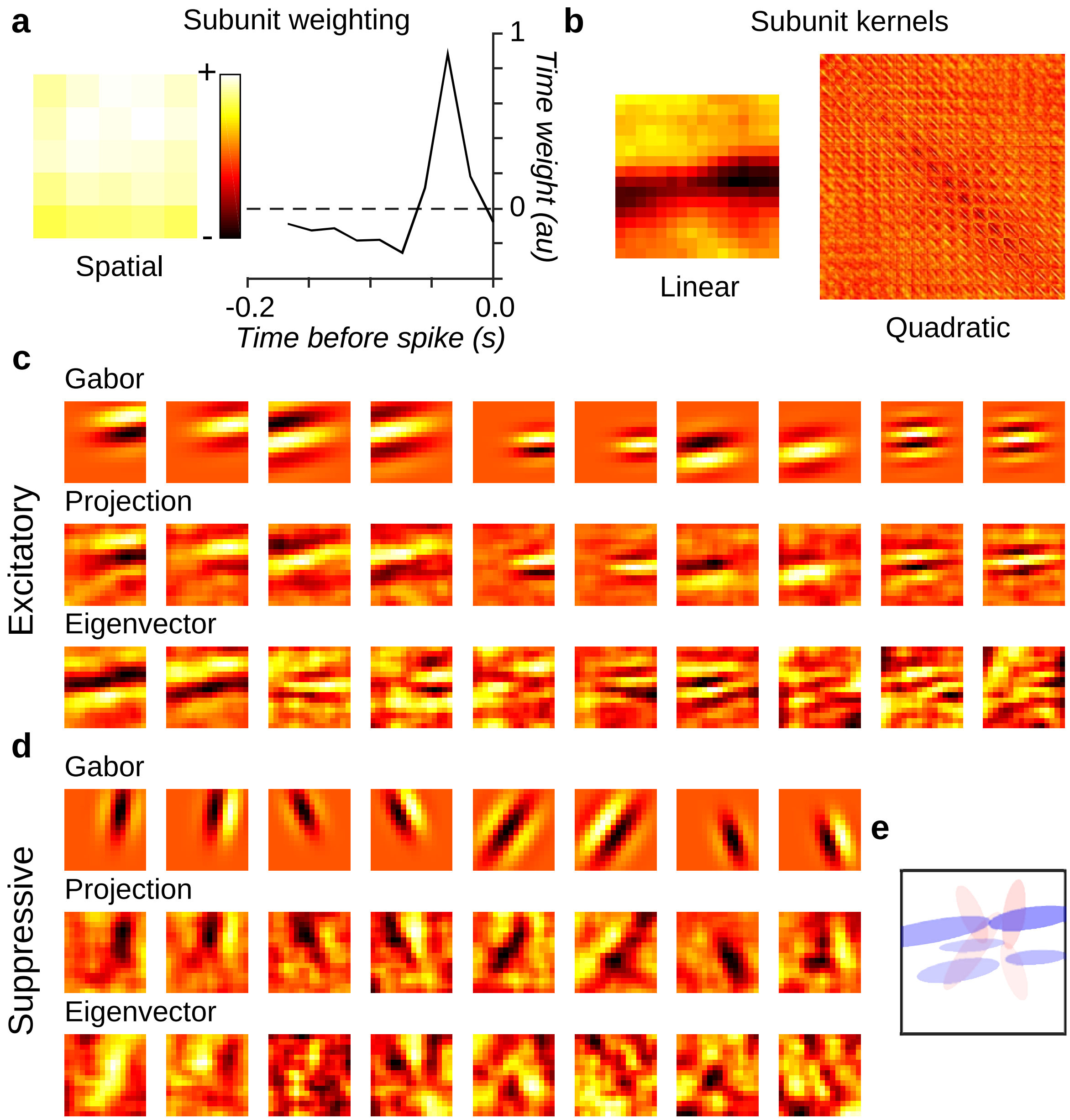

Supplementary Figure 6: **Full analysis of an example V2 cell. a.** The subunit weighting $\mathbf{v}^{(2)}$ decomposed into the primary spatial and temporal components using singular value decomposition (SVD). **b.** The linear ($\mathbf{v}^{(1)}$) and quadratic kernel (J) of the subunits. **c.** The excitatory components of J. The top row shows the pairs of Gabors fit to the excitatory part of J. **d.** Same as **c** but for suppressive components. **e.** The excitatory (blue) and suppressive (red) components overlaid on each other. Each ellipse represents one pair of Gabors. The relative opacity of the ellipses indicates the relative weight of the Gabors. The combination reveals selectivity for horizontal curves and suppression in the presence of vertical lines. Neuron e0040.

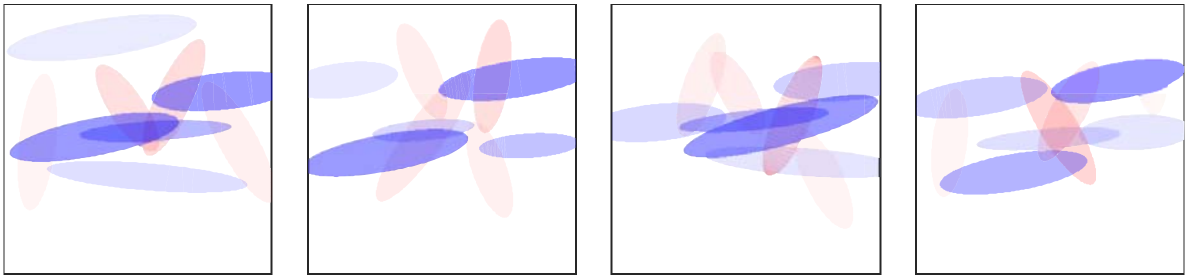

Supplementary Figure 7: **Gabors fits to different jackknifes show consistency of preferred stimuli.** The algorithm consistently finds excitation by horizontal lines and suppressive by vertical lines.

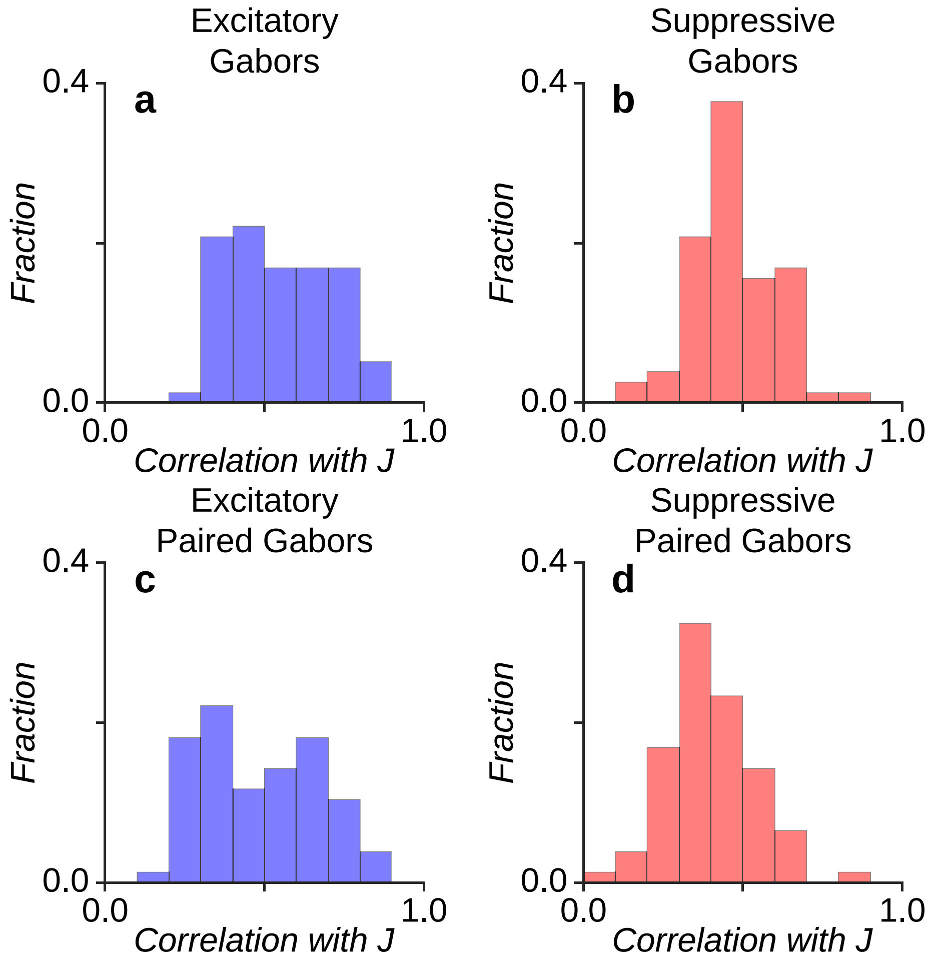

Supplementary Figure 8: **Gabor wavelets describe the structure of the quadratic kernel. a.** The distribution of the correlations between the significant excitatory portion of J and the approximation using individual Gabor wavelets. **b.** Same as **a** but for the suppressive part of J. **c.** The correlations between the excitatory part of J and the approximation using pairs of Gabor wavelets. **d.** Same as **c** but for the suppressive part of J. The paired Gabor approximation performs almost as well despite having less than half as many parameters.

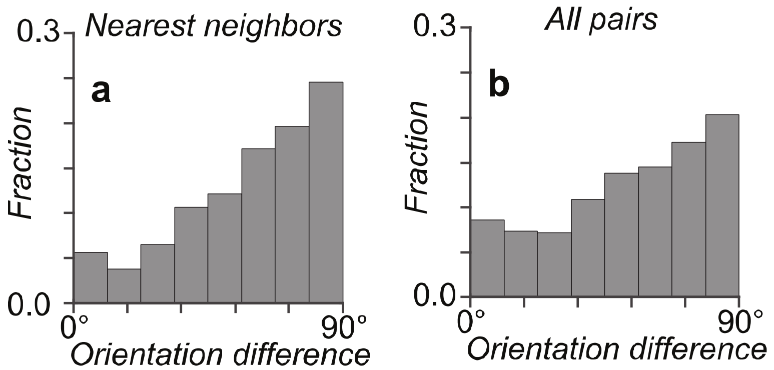

Supplementary Figure 9: **Cross-orientation suppression in V2 neurons. a.** The distribution of nearest-neighbor differences between excitatory and suppressive features for all V2 neurons in the dataset (both classes combined). **b.** Same as **a** but expanded to include all excitatory-suppressive pairs, not just nearest neighbors.

Supplementary Note 1

Test of the algorithm on model neurons

To illustrate and test how the reconstruction algorithm works in practice, we created a model neuron whose response properties mimicked the salient aspects of neural responses in area V2.

The model included graded position invariance as well as selectivity to combinations of Gabor features at each retinotopic location. The Gabor features were included in pairs where all Gabor parameters were identical for the two features forming a pair except for the spatial phase, which differed by 90°. Two Gabors pairs were excitatory and one Gabor pair was suppressive, cf. Supplementary Fig. 1. The two pairs of excitatory features had identical parameters except for a spatial offset and had slightly different orientations such that they combined to form a curved line. The suppressive pair was centered over the intersection of the excitatory pair with an orientation orthogonal to the mean orientation of the excitatory pair. The joint action of these features can be summarized by plotting the resulting quadratic kernel J, shown in panel c. The steps involved in the construction of the quadratic kernel are schematized in panels a and b. The relative contributions of each grid location to the model neuron's firing rate was weighted according to a circularly symmetric Gaussian with a standard deviation of 1.75 pixels.

The reconstruction algorithm successfully characterized the feature selectivity and invariance properties of our model V2 neuron. By diagonalizing J and determining the number and magnitude of eigenvalues that make statistically significant contribution to it (see above subsection on *Eigenvector significance* for details), the algorithm correctly identified that the model neuron was selective for a 6-D stimulus space. Further, the algorithm correctly identified that four of these features were excitatory and two were suppressive based on the sign of the corresponding contributions (panel d). The subspace formed by the relevant features was also correctly identified, because the orthogonal representations of both the model and reconstructed features closely resembled each other, cf. Supplementary Fig. 1 e, h. The average reconstruction per dimension was 0.9578 ± 0.0019. The correlation in the firing rate

between the true and reconstructed model was 0.956 ± 0.005. As a final step, we were also able to successfully recover the actual relevant features underlying the response properties of this model neuron. Because the Gabor features of the model are not orthogonal to each other, their orthogonal representation shown in panel h deviates from the original features shown in panel g. Fitting the reconstructed quadratic kernel J (from panel c) as arising from combinations of Gabor pairs, we were able to match the original Gabors shown in Supplementary Fig. 1 g up to the spatial phase of the Gabors. [The spatial phase of Gabor does not affect the neural firing because of the squaring nonlinearity and thus does not represent a relevant parameter of the model].

In Supplementary Fig. 2 we show how reconstruction quality is expected to change as a function of dataset size. Overall, reconstruction remains robust across two orders of magnitude in dataset size.

To further test the algorithm, we verified that cross-orientation suppression does not inherently follow from the optimization procedure (Supplementary Fig. 3) We applied the reconstruction algorithm to the responses of a model neuron whose suppressive features did not have orthogonal Gabor orientation to the orientation of its excitatory features. The correct reconstruction of the relevant subspace by the algorithm (subspace projection of 0.957) indicates that cross-orientation suppression observed for V2 responses is not induced by the optimization procedure.

Supplementary References:

1. Rowekamp, R.J. & Sharpee, T.O. Analyzing multicomponent receptive fields from neural responses to natural stimuli. *Network* **22**, 1-29 (2011).
